# Predicting Key Genes and Therapeutic Molecular Modelling to Explain the Association between *Porphyromonas gingivalis* (*P. gingivalis*) and Alzheimer’s Disease (AD)

**DOI:** 10.3390/ijms24065432

**Published:** 2023-03-12

**Authors:** Ahmed Hamarsha, Kumarendran Balachandran, Ahmad Tarmidi Sailan, Nurrul Shaqinah Nasruddin

**Affiliations:** Department of Craniofacial Diagnostics and Biosciences, Faculty of Dentistry, University Kebangsaan Malaysia, Jalan Raja Muda Abdul Aziz, Kuala Lumpur 50300, Malaysia

**Keywords:** Alzheimer’s disease, *Porphyromonas gingivalis*, bioinformatics

## Abstract

The association between *Porphyromonas gingivalis* (*P. gingivalis*) and Alzheimer’s disease (AD) remains unclear. The major aim of this study was to elucidate the role of genes and molecular targets in *P. gingivalis*-associated AD. Two Gene Expression Omnibus (GEO) datasets, GSE5281 for AD (n = 84 Alzheimer’s, n = 74 control) and GSE9723 (n = 4 *P. gingivalis*, n = 4 control), were downloaded from the GEO database. Differentially expressed genes (DEGs) were obtained, and genes common to both diseases were drawn. Additionally, Kyoto Encyclopedia of Genes and Genomes (KEGG) and Gene Ontology (GO) analysis was performed from the top 100 genes (50 upregulated and 50 downregulated genes). We then proceeded with CMap analysis to screen for possible small drug molecules targeting these genes. Subsequently, we performed molecular dynamics simulations. A total of 10 common genes (CALD1, HES1, ID3, PLK2, PPP2R2D, RASGRF1, SUN1, VPS33B, WTH3DI/RAB6A, and ZFP36L1) were identified with a *p*-value < 0.05. The PPI network of the top 100 genes showed UCHL1, SST, CHGB, CALY, and INA to be common in the MCC, DMNC, and MNC domains. Out of the 10 common genes identified, only 1 was mapped in CMap. We found three candidate small drug molecules to be a fit for PLK2, namely PubChem ID: 24971422, 11364421, and 49792852. We then performed molecular docking of PLK2 with PubChem ID: 24971422, 11364421, and 49792852. The best target, 11364421, was used to conduct the molecular dynamics simulations. The results of this study unravel novel genes to *P. gingivalis*-associated AD that warrant further validation.

## 1. Introduction

Alzheimer’s disease (AD) is characterised as a progressive neurological disorder that impacts thinking, behaviour, and memory [1]. The etiology of AD is poorly understood [2]. Many studies have found that AD is linked with genetics. Increasingly, much evidence has shown that AD can also be caused by environmental risk.

Many bacteria, including *Streptococcus mutans* and *Actinomyces viscosus*, have been associated with an increased risk of AD development via modifying gene expression [3]. Studies found that infection with the herpes simplex virus 1 (HSV-1) could lead to changes in gene expression in human brain cells that were similar to those observed in the brains of Alzheimer’s patients [4]. The researchers suggested that these changes could contribute to the development of AD. However, it is not yet clear exactly how microbes might be capable of altering genes in the brain, but one possibility is that they may be able to trigger changes in the epigenome, which is the set of chemical modifications to DNA and associated proteins that regulate gene expression [5]. Some studies have suggested that infection with certain microbes may lead to changes in the epigenome that could contribute to the development of AD [6]. While the exact mechanisms by which microbes might alter genes in the brain are not yet fully understood, there is growing evidence to suggest that this could be one way in which these microbes contribute to the development and progression of AD [7]. Additionally, microbes may induce changes in the epigenome through a process called DNA methylation. DNA methylation is the addition of a methyl group to DNA, which can alter gene expression [8]. Some studies have suggested that infection with certain microbes may lead to changes in DNA methylation patterns in the brain, which could contribute to the development of AD [9].

More recently, studies have found that *Porphyromonas gingivalis (P. gingivalis)*, a Gram-negative bacterium, is localised in the brain of humans with AD [10]. According to recent research, *P. gingivalis* can lead to systemic inflammation and is associated with other chronic inflammatory disorders such as rheumatoid arthritis and cardiovascular disease [11]. *P. gingivalis* has also been connected to systemic diseases such as endocarditis and sepsis [12].

Gene expression profiling using microarray and sequencing technologies has become a common tool in research and has the potential to provide a more comprehensive understanding of the molecular mechanisms underlying these diseases [13]. Multiple gene expression studies on AD have been conducted by different researchers, resulting in the availability of a large number of gene expression datasets [14]. By integrating these datasets, it is possible to identify key genes that are involved in the development and prognosis of *P. gingivalis*-associated AD [15].

Therefore, in this study, gene expressions of AD and *P. gingivalis* were downloaded from Gene Expression Omnibus (GEO) database. We identified differentially expressed genes (DEGs) by bioinformatic tools and identified DEGs related to AD and *P. gingivalis.* Additionally, we utilised clustering tools to identify hub genes using Gene Ontology (GO) enrichment analysis, Kyoto Encyclopedia of Genes and Genomes (KEGG), and protein–protein network interaction (PPI) analysis. Hub genes identified from this study provide insights into the molecular mechanism to elucidate the association between AD and *P. gingivalis.*

## 2. Results

### 2.1. Data Normalisation 

**Figure 1:**
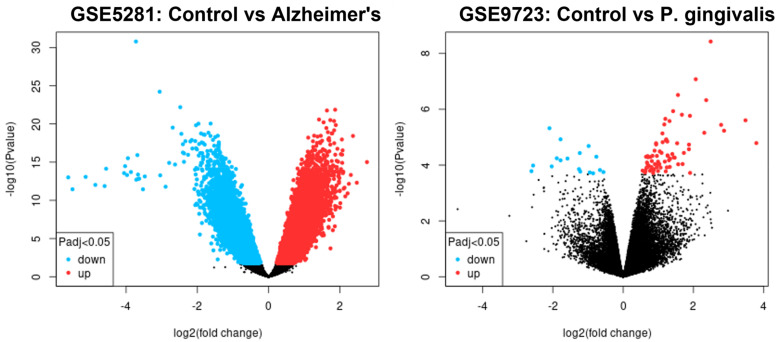
Volcano plots showing the upregulated and downregulated genes in GSE5281 and GSE9723.

**Figure 2:**
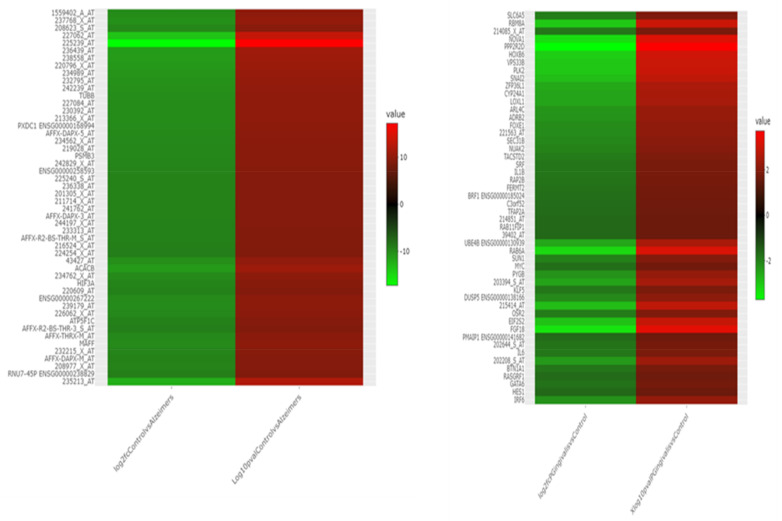
Heat maps of the differentially expressed genes (DEGs) according to their genes and expressions. The gene symbols are shown on the left side of the heat maps.

R 4.2.1 software was used to analyse the original data. Firstly, quality was evaluated, data were normalised, and the expression density is shown in Figures S1 and S2 (Supplementary Data). We found 2540 upregulated and 1776 downregulated in GSE 5281. A total of 39 were upregulated and 11 downregulated in GSE9723, as shown in Figure 1. These DEGs are shown by volcano plots in Figure 2. The volcano plots shown identify genes that are *p* < 0.05 and are used as criteria for significance. The heat maps (Figure 2) show the hierarchical clustering of the DEGs.

### 2.2. DEGs Common to AD and P. gingivalis

**Figure 3:**
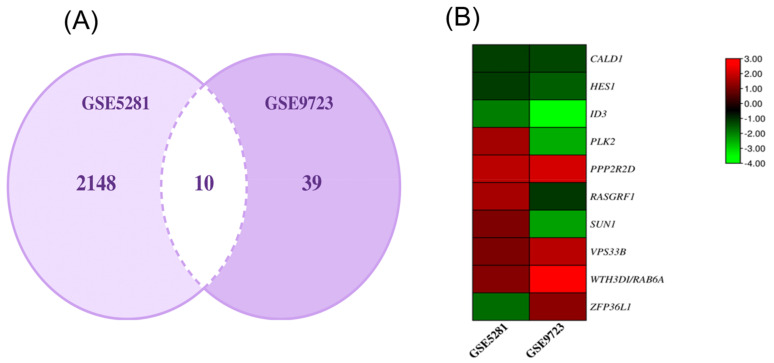
(**A**) Common genes and (**B**) heat map of *P. gingivalis*-associated AD.

Using R, we found the common genes associated with datasets GSE5281 and GSE9723. The 10 genes were CALD1, HES1, ID3, PLK2, PPP2R2D, RASGRF1, SUN1, VPS33B, WTH3DI/RAB6A, and ZFP36L1. We then built a heat map to show the relativity of these found genes. Both of these findings are depicted in Figure 3.

### 2.3. GO and KEGG Analysis 

**Figure 4:**
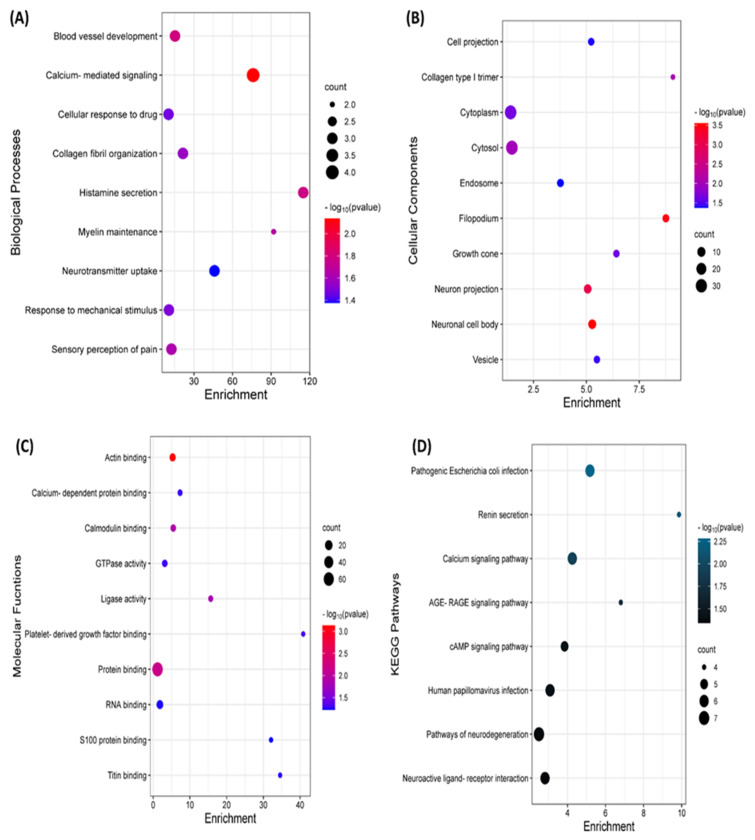
Gene Ontology and Kyoto Encyclopedia of Genes and Genomes (KEGG) analysis of the differentially expressed genes (DEGs) according to (**A**) biological processes (BPs), (**B**) cellular components (CCs), (**C**) molecular functions (MFs), and (**D**) KEGG pathways.

To elucidate the role of these genes, a Gene Ontology (GO) and Kyoto Encyclopedia of Genes and Genomes (KEGG) pathway was constructed. Most of the functions of the common genes were involved in calcium-mediated signalling, actin binding, filopodium, and some involvement in pathogenic E. coli infection. The summary of this finding is presented in Figure 4.

### 2.4. PPI Network and Hub Gene Selection 

**Figure 5:**
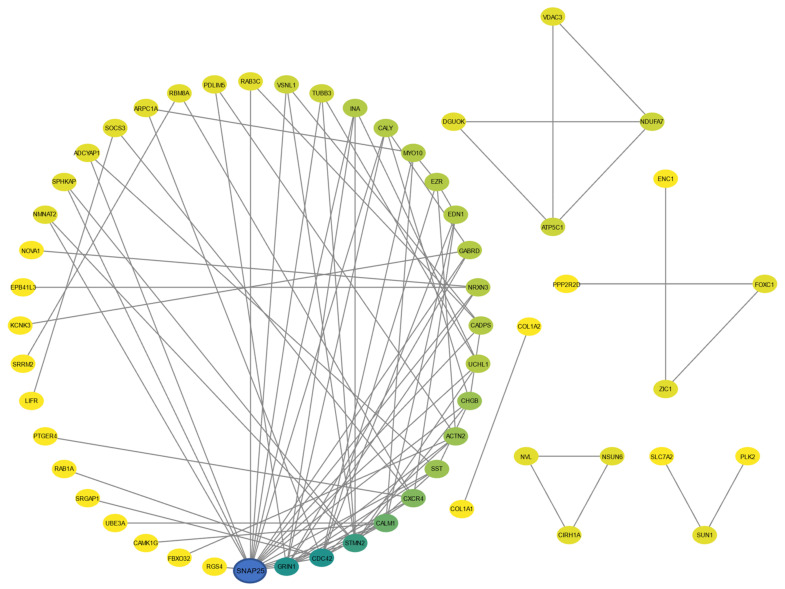
Protein–protein interaction (PPI) network from top 100 genes. Proteins are denoted as nodes and interactions as edges.

The PPI network from 10 identified common genes showed limited interaction. We then selected the top 100 genes (top 50 upregulated and top 50 downregulated) for our PPI network. The nodes indicate proteins, and the edges indicate their interactions, as shown in Figure 5. The specific functions of the genes and their indications are provided in Supplementary File S1.

### 2.5. CMap and Molecular Docking 

**Figure 6:**
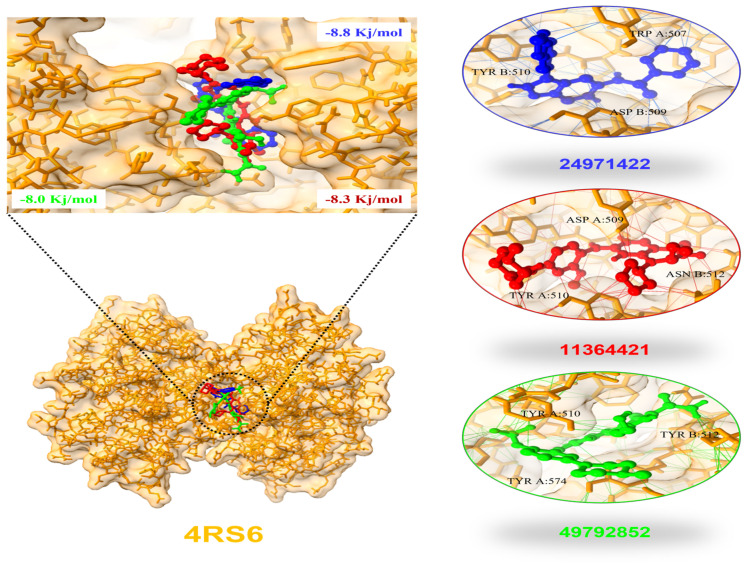
Molecular docking of *PLK2* with 3 drug targets.

Our CMap analysis only yielded one result of the ten common genes screened. PLK2 was then chosen for further analysis. A total of three drug targets were identified to exert biological changes to protein PLK2. They are summarised in Table 1, and the results of molecular docking are shown in Figure 6.

### 2.6. Molecular Dynamic Simulations

**Figure 7:**
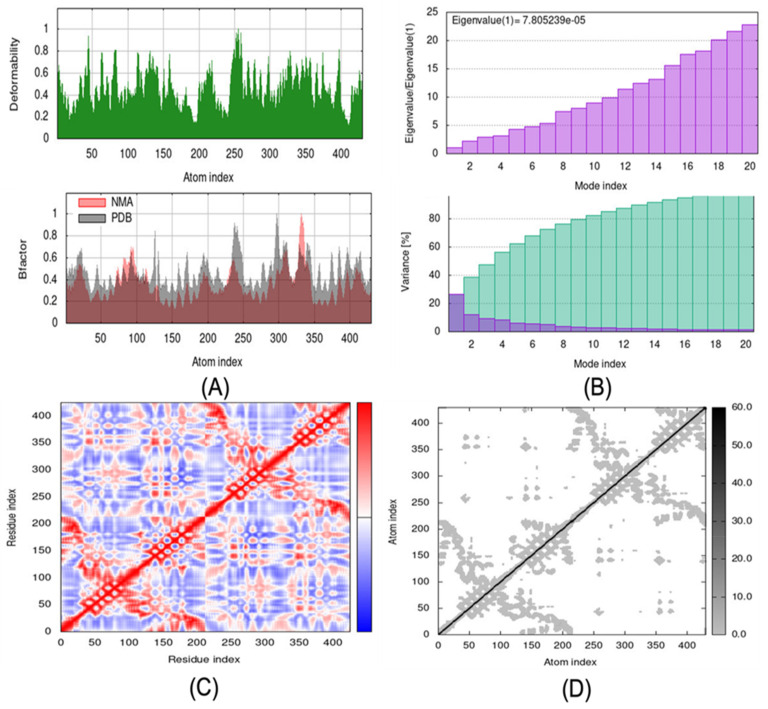
Results of PLK2-11364421 molecular dynamics simulations using iMODS: (**A**) deformability and B-factor plots; (**B**) eigenvalue and variance plots (Colored bars show the individual (purpule) and cumulative (green) variances.); (**C**) elastic network model; and (**D**) covariance map.

The best docking complex was chosen (PubChem ID: 11364421). The mobility characteristics of docked proteins are determined by deformability and B-factor. The deformability and B-factors of the PLK2 and 11364421 complexes show the peaks corresponding to deformable regions in the proteins, with the greatest peaks representing high deformability regions (Figure 7A). Figure 7B shows the eigenvalue and variance graphs of the PLK2 and 11364421 complexes. The variation graph of 11364421 with the target PLK2 shows individual variance with purple-shaded bars and cumulative variance with green-shaded bars. The complex covariance matrix depicts the correlations between residues in a complex. The red colour in Figure 7C in the matrix represents a good correlation between residues, while the white colour represents uncorrelated motion. Furthermore, the blue tint indicates anticorrelations. The higher the correlation, the more complicated the system. The docked proteins’ elastic maps (Figure 7D) show the atoms’ connections, with darker grey areas indicating stiffer regions.

## 3. Discussion

### 3.1. Common Genes Found in P.gingivalis and AD

CALD1 (Calcium-binding protein 1) is a protein that is encoded by the CALD1 gene in humans. It is a member of the calcyphosine family of calcium-binding proteins and is expressed in various tissues, including the brain. Some research has suggested that CALD1 may be involved in the development of AD, although more research is needed to fully understand its role in this condition [16]. In a particular study, it was found that CALD1 expression was significantly increased in the brains of people with AD compared to those without the condition and that CALD1 may be involved in the production of amyloid beta, a protein that is believed to be a key contributor to the development of AD [17,18]. Some research has suggested that CALD1 may be involved in the development of periodontitis, a type of gum disease characterised by inflammation and loss of the tissue and bone that support the teeth [19,20]. It was found that CALD1 expression was significantly increased in the gingiva of people with periodontitis compared to those without the condition and that CALD1 may be involved in the immune response to periodontitis.

The HES1 protein is a member of the family of transcription factors known as basic helix–loop–helix (bHLH) factors, and it functions as a transcriptional repressor for genes whose transcription is dependent on the bHLH protein. The protein attaches to the N-box promoter region rather than the typical enhancer box because it has a particular type of basic domain with a helix-interrupting protein (E-box). According to a study, HSE1 Melatonin’s protective effect on soluble A1-42-induced memory impairment, astrogliosis, and synaptic dysfunction in the rat hippocampus through the Musashi1/Notch1/Hes1 signalling pathway [21]. According to certain studies, HES1 may contribute to the onset of oral cancer cells sustainably infected with *P. gingivalis* exhibit resistance to Taxol and have a higher metastatic potential [22].

The human ID3 gene produces the DNA-binding protein inhibitor ID-3 protein [23]. Helix–loop–helix (HLH) proteins belonging to the ID family lack a fundamental DNA-binding domain and suppress transcription by forming dimers that are ineffective at binding to DNA. Regarding histone H3K9me3-based epigenome signatures, research has indicated that ID3 is connected with synaptic impairment in AD [24]. In one study conducted, gene expression changes in the various functional categories related to periodontitis in adults and aged animals the ID3 were decreased with periodontitis [25].

Protein kinase serine/threonine, the PLK2 gene in humans, codes for the enzyme PLK2. The ‘polo’ family of serine/threonine protein kinases, which includes serum-inducible kinase, is involved in healthy cell division. They discovered that PLK2 activity inhibition alters APP and tau pathology and enhances synaptic content in a sex-dependent manner in Alzheimer’s dementia, suggesting that it may contribute to the aetiology of the illness [26]. A study has shown that PLK2’s significance in cancer is somewhat debatable; evidence points to both an oncogenic and a tumour suppressor role in a variety of malignancies [27].

The PPP2R2D gene in humans encodes the protein known as PP2A subunit B isoform delta, often referred to as serine/threonine-protein phosphatase 2A 55 kDa regulatory subunit B delta isoform. There is no research on this gene’s relationship to *P. gingivalis* or AD.

A nuclear envelope protein with an UNC84 (SUN) domain is encoded by the protein-coding gene SUN1 (Sad1 and UNC84 domain containing 1). This gene belongs to the unc-84 homolog family. The protein aids in nuclear migration and anchoring. Spliced transcript variations have also been reported as an alternative (provided by RefSeq, January 2019). A previous study found that the accumulation of the inner nuclear envelope protein Sun1 is pathogenic in progeroid and dystrophic laminopathies, which results in AD [28]. No study has shown SUN1-gene-associated *P. gingivalis*.

VPS33B (vacuolar protein sorting-associated protein 33B) is a protein that, in humans, is encoded by the VPS33B gene. The translational profile of striatopallidal neurons is preferentially altered by deep brain stimulation of the subthalamic nucleus in an animal model of Parkinson’s disease, and one of the genes they identified was VPS33B [29]. By integrating transcriptome analysis, the gene VPS33B is implicated in the different gene expression traits in the interactions between epithelial cells and *P. gingivalis* [30].

WTH3DI/RAB6A is a type of protein coding; members of the small GTPase superfamily’s RAB family are encoded by this gene. The targeting and fusing of transport carriers to acceptor compartments are regulated by the binding of GTPases of the RAB family to different effectors. This protein is found at the Golgi apparatus, which controls both retrograde and forward trafficking provided by HUGO Gene Nomenclature. No study was found related to this gene-associated AD and *P. gingivalis*.

**Figure 8:**
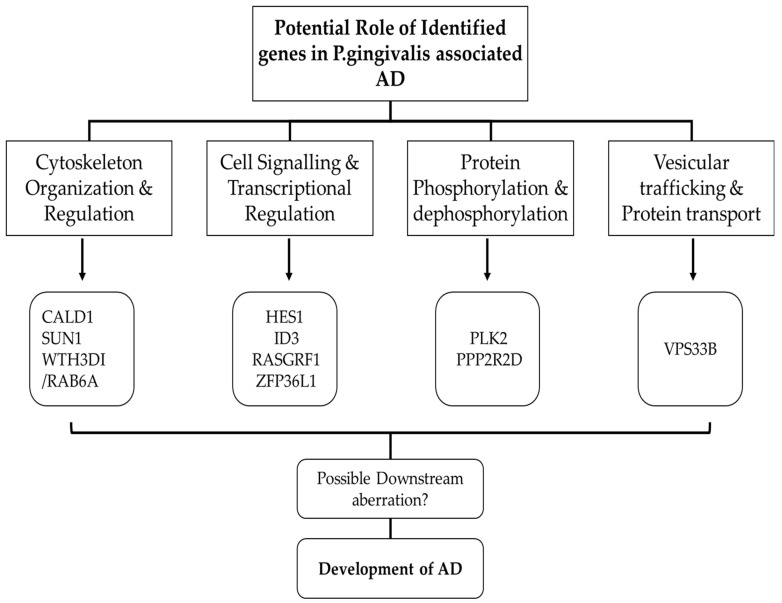
The functional characterisation of identified common genes.

The ZFP36L1 gene belongs to the TIS11 family of early response genes, which are activated by a variety of agonists, including the polypeptide mitogen EGF and the phorbol ester TPA. This gene is highly conserved between species and features motifs found in other early-response genes in its promoter. A distinctive putative zinc finger domain with a recurring cys-his pattern can be found in the encoded protein. Most likely, this putative nuclear transcription factor controls how the body reacts to growth stimuli. This gene has been associated with a variety of alternatively spliced transcript variants that encode distinct isoforms (provided by RefSeq, September 2011). Characterisation of ZFP36L1 in the context of multiple sclerosis and functional immunological effects connected to the susceptibility to the disease, according to a study [31]. APN impairs the ability of macrophages, which play a significant role in periodontitis, to function. Through dependent signalling pathways, APN first stimulates the synthesis of TNF-α, which increases the expression of IL-10 and, as a result, reduces the inflammatory response of macrophages exposed to LPS [32]. Moreover, by promoting macrophage autophagy, APN can also reduce the expression of inflammatory mediators brought on by LPS. APN increases the production of ZFP36L1, which inhibits the interaction between Bcl-2 and Beclin-1 and, in turn, stimulates Beclin-1-activated autophagy in macrophages by destabilizing the mRNA of Bcl-2 [33]. A summary of the functions of the genes and possible roles is provided in Figure 8.

### 3.2. PPI Network Selected Hub Genes UCHL1, SST, CHGB, CALY, and INA

Ubiquitin carboxy-terminal hydrolase L1 (UCHL1) belongs to a gene family whose members hydrolyze short C-terminal ubiquitin adducts to produce the ubiquitin monomer [34]. Highly specialised neurons, diffuse neuroendocrine system cells, and their tumours express UCHL1 [35]. It is known to play a role in AD [36]. It is thought that UCHL1 may be involved in the clearance of proteins that accumulate in the presence of AD, such as amyloid-beta [37]. UCHL1 has been identified as a potential marker for periodontitis [38]. The presence of UCHL1 in periodontitis may be associated with increased inflammation and tissue destruction [39]. UCHL1 may be a potential target for the treatment of AD and periodontitis [40]. Somatostatin is present throughout the body and binds to high-affinity G-protein-coupled somatostatin receptors to prevent the production of multiple secondary hormones. Through its interactions with thyroid stimulating hormone and pituitary growth hormone, this hormone plays a key role in the regulation of the endocrine system (provided by RefSeq, July 2008). It is also believed to play a role in the progression of AD [41]. Studies have shown that somatostatin levels are significantly lower in individuals with AD and that this decrease is associated with increased levels of the amyloid beta peptide, which is known to be a major cause of AD [42]. Somatostatin has been found to play a role in periodontitis, or inflammation of the gums; it is believed that somatostatin may be involved in the inflammatory process that leads to periodontitis [43]. Secretogranin-1, also known as Chromogranin B, is a protein that the CHGB gene in humans codes for; it is a member of the grain protein family. Chromogranin B is a gene that has been associated with AD. Studies have shown that individuals with AD have higher levels of CHGB in their brains than those without the disease. It is believed that CHGB may be involved in the accumulation of tau proteins, which are associated with AD, and the formation of amyloid plaques, which are also associated with the disease [44]. Currently, there is no evidence to suggest that CHGB, or Chromogranin B, is involved in periodontitis. However, research into this gene and its role in other inflammatory diseases may provide insight into its potential role in periodontitis. Neuron-specific vesicular protein is a type II single transmembrane protein that is expressed by the CALY gene in humans. It is necessary for the maximum accelerated calcium release upon stimulation of purinergic or muscarinic receptors [45]. CALY has been associated with AD. Studies have shown that levels of Calcyon are significantly lower in individuals with AD than in those without the disease [46]. It is believed that Calcyon may be able to regulate the activity of certain proteins that are involved in the progression of AD [47]. Currently, there is no evidence to suggest that neuron-specific vesicular protein Calcyon is involved in periodontitis. Alpha-internexin is a type of class IV intermediate filament with a mass of 66 kDa. The rat spinal cord and optic nerve were originally used to purify the protein [48]. A comparable central rod domain found in alpha-internexin includes about 310 amino acid residues and forms a highly conserved alpha-helical region. An area called the amino-terminal head, and a region called the carboxy-terminal tail, surround the core rod domain, which is in charge of the coiled-coil structure [49]. INA encodes neuronal intermediate filament protein found mostly in the neurons of the nervous system during early development [50]. No study has shown a relationship between the INA gene and periodontitis.

### 3.3. Molecular Docking of PLK2 with 11364421 (C_28_H_39_N_7_0_3_)

We used iMODS to evaluate and define the flexibility of PLK2 with 11364421. We hypothesize that based on the conceivable interactions of the identified proteins with PLK2, they can serve as prospective therapeutic candidates and targets to attenuate the pathological process in *P. gingivalis*-associated AD.

## 4. Materials and Methods

### 4.1. Pipeline of Research

**Figure 9:**
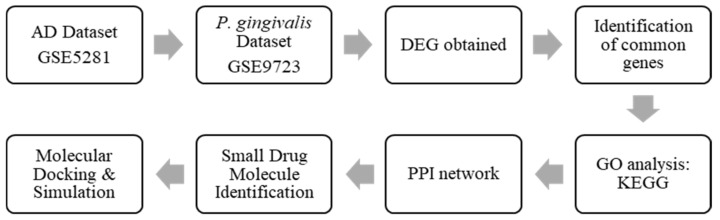
Flow chart of this study.

The flowchart of the conduct of this study is summarised in Figure 9.

### 4.2. Data Acquisition

The National Centre for Biotechnology Information (NCBI) produced and maintains the gene expression database known as the GEO database. It includes high throughput gene expression data provided by international research institutions [51]. To determine the molecular mechanism of the occurrence and development of *P. gingivalis*-associated AD, two microarray datasets from the GEO database, namely, GSE5281 and GSE9723, were downloaded. The former was a study conducted on Alzheimer’s patients, and the latter was using cells infected with *P. gingivalis.* The data’s executive summary is presented in Table 2 below.

### 4.3. DEGs Screening

Using the limma package in R, we identified the differentially expressed genes (DEGs) between AD and *P. gingivalis*. The criteria of *p* < 0.05 and log2FC > 1 were set before entering to VENN tool to visualise the intersecting genes.

### 4.4. Functional Analysis of Common Genes

The Database for Annotation, Visualization, and Integrated Discovery (DAVID) web tool is used to carry out GO enrichment analysis and KEGG pathway analysis [55]. The three categories of GO analysis, which include biological processes (BPs), cellular components (CCs), and molecular functions (MFs), were performed [56]. A *p*-value < 0.05 is considered significant.

### 4.5. Construction of PPI Network

A protein–protein interaction (PPI) analysis was carried out using the STRING database to investigate the relationships between common genes (https://string-db.org/) (accessed on 26 December 2022). All known and anticipated protein–protein interactions, including both physical and details functional relationships, are listed in the STRING database [57]. The minimum interaction score was set to 0.400 as a criterion for statistical significance. The PPI is illustrated by the nodes as proteins and lines as interactions.

### 4.6. CMap Analysis, Molecular Docking, and Simulation

We will be predicting small drug molecules using CMap in accordance with specific gene expression signatures offered by the database. A small drug molecule with a negative mean score may be able to reverse biological effects and so have potential therapeutic utility (*p* < 0.05 is considered significant). The effectiveness and binding potential were assessed using molecular docking. Firstly, crystal structures are obtained from RCSB Protein Data Bank (PDB, http://www.rcsb.org) (accessed on 26 December 2022), and the mol2 file formats of the compounds were retrieved from the PubChem database. PyMOL 2.3.1 was used to dehydrate target proteins and remove ligands. AutoDock Tool 1.5.6 software was used to hydrogenate, calculate its charge, and store it in PDBQT format. PyRx and Autodock Vina v1.2.0 software was used to visualize the findings of molecular docking. The iMOD server iMOD server (iMODS) (http://imods.chaconlab.org) (accessed on 26 December 2022) was used to run molecular dynamics simulations to calculate the stability and molecular mobility of the bound protein-113644421 complexes. In addition to calculating molecular mobility, iMODS was employed to analyse the structural dynamics of the docking complexes. The elastic network, deformability, B-factor, eigenvalues, variance, and covariance map were all used to show how stable the protein-11364421 complexes were. All parameters for the docked PDB files used as input were left at default when they were uploaded to the iMODS server.

## 5. Conclusions

In this study, 10 crossover genes were identified, indicating a potential association between AD and *P. gingivalis* as the key pathogen in periodontitis. Hence, one potential therapeutic target was identified for *P. gingivalis*-induced-AD treatment.

However, further in vivo and in vitro studies are needed to confirm these findings.

## Figures and Tables

**Table 1:** Chemical structure and ID according to CMap for *PLK2* targets.

ID	Chemical Structure	IUPAC Name
24971422	C_21_H_17_N_3_O_3_	3-(1,3-benzodioxol-5-yl)-N-[(1S)-1-phenylethyl]-[1,2] oxazolo [5,4-c] pyridin-5-amine
11364421	C_28_H_39_N_7_O_3_	4-[[(7R)-8-cyclopentyl-7-ethyl-5-methyl-6-oxo-7H-pteridin-2-yl] amino]-3-methoxy-N-(1-methylpiperidin-4-yl) benzamide
49792852	C_24_H_27_F_3_N_8_O_3_	1-(2-hydroxyethyl)-8-[5-(4-methylpiperazin-1-yl)-2-(trifluoromethoxy) anilino]-4,5-dihydropyrazolo[4,3-h] quinazoline-3-carboxamide

**Table 2:** The summary of the datasets collected from the Gene Expression Omnibus (GEO) database.

Reference	GEO Series	Platform	Control (N)	Effected (N)	Country
Readhead et al., 2018 [52], Liang et al., 2007 [53]	GSE5281	GPL570	74	87	USA
Handfield et al., 2005 [54]	GSE9723	GPL96	4	4	USA

## Data Availability

Not Applicable.

## Supplementary Materials

The following supporting information can be downloaded at: https://www.mdpi.com/article/10.3390/ijms24065432/s1.
